# Supplementary material for: Factor analysis for the clustering of cardiometabolic risk factors and sedentary behavior, a cross-sectional study
Source: PLoS One. 2020 Nov 16;15(11):e0242365. doi: 10.1371/journal.pone.0242365 (PMC7668610; doi:10.1371/journal.pone.0242365)
Supplement: S1 Table — (DOCX) [file pone.0242365.s001.docx]

**S1 Table. Factor analysis in patients <65 years of age**

|  | Component | | | | |
| --- | --- | --- | --- | --- | --- |
|  | 1 | 2 | 3 | 4 | 5 |
| Waist | 0.813 |  |  |  |  |
| BMI | 0.802 |  |  |  |  |
| HDL | −0.735 |  |  |  |  |
| Uric acid | 0.677 |  |  |  |  |
| Triglyceride | 0.518 |  |  |  |  |
| hsCRP | 0.463 |  |  |  |  |
| Cholesterol |  | 0.984 |  |  |  |
| LDL |  | 0.944 |  |  |  |
| GLU |  |  | 0.913 |  |  |
| A1c |  |  | 0.903 |  |  |
| SBP |  |  |  | 0.916 |  |
| DBP |  |  |  | 0.900 |  |
| METs (weekly) |  |  |  |  | 0.794 |
| Sitting time (minutes) |  |  |  |  | −0.690 |
| Eigen values | 2.928 | 1.940 | 1.919 | 1.858 | 1.148 |
| Rotation Sums of Squared Loadings (% of Variance) | 20.914 | 13.856 | 13.706 | 13.271 | 8.202 |
| Rotation Sums of Squared Loadings (Cumulative %) | 20.914 | 34.770 | 48.476 | 61.747 | 69.948 |
| BMI= body mass index;GLU=serum glucose; HDL=high density lipoprotein; LDL=low density lipoprotein; SBP=systolic blood pressure; DBP=diastolic blood pressure; MET= metabolic equivalent; HbA1C=hemoglobin A1C. | | | | | |
