# Supplementary material for: Factor analysis for the clustering of cardiometabolic risk factors and sedentary behavior, a cross-sectional study
Source: PLoS One. 2020 Nov 16;15(11):e0242365. doi: 10.1371/journal.pone.0242365 (PMC7668610; doi:10.1371/journal.pone.0242365)
Supplement: S2 Table — (DOCX) [file pone.0242365.s002.docx]

**S2 Table. Factor analysis in patients >65 years of age**

|  | Component | | | | |
| --- | --- | --- | --- | --- | --- |
|  | 1 | 2 | 3 | 4 | 5 |
| Waist | 0.756 |  |  |  |  |
| BMI | 0.742 |  |  |  |  |
| HDL | −0.687 |  |  |  |  |
| Uric acid | 0.633 |  |  |  |  |
| Triglyceride | 0.552 |  |  |  |  |
| hsCRP |  |  |  |  |  |
| Cholesterol |  | 0.975 |  |  |  |
| LDL |  | 0.952 |  |  |  |
| GLU |  |  | 0.922 |  |  |
| A1c |  |  | 0.915 |  |  |
| SBP |  |  |  | 0.883 |  |
| DBP |  |  |  | 0.879 |  |
| METs (weekly) |  |  |  |  | −0.658 |
| Sitting time (minutes) |  |  |  |  | 0.632 |
| Eigen values | 2.533 | 2.021 | 1.792 | 1.734 | 1.220 |
| Rotation Sums of Squared Loadings (% of Variance) | 18.096 | 14.434 | 12.801 | 12.383 | 8.714 |
| Rotation Sums of Squared Loadings (Cumulative %) | 18.096 | 32.530 | 45.331 | 57.713 | 66.427 |
| BMI= body mass index;GLU=serum glucose; HDL=high density lipoprotein; LDL=low density lipoprotein; SBP=systolic blood pressure; DBP=diastolic blood pressure; MET= metabolic equivalent; HbA1C=hemoglobin A1C. | | | | | |
