# Supplementary material for: Factor analysis for the clustering of cardiometabolic risk factors and sedentary behavior, a cross-sectional study
Source: PLoS One. 2020 Nov 16;15(11):e0242365. doi: 10.1371/journal.pone.0242365 (PMC7668610; doi:10.1371/journal.pone.0242365)
Supplement: S3 Table — (DOCX) [file pone.0242365.s003.docx]

**S3 Table. Factor analysis in male patients**

|  | Component | | | | |
| --- | --- | --- | --- | --- | --- |
|  | 1 | 2 | 3 | 4 | 5 |
| BMI | 0.848 |  |  |  |  |
| Waist | 0.837 |  |  |  |  |
| HDL | −0.611 |  |  |  |  |
| Uric acid | 0.498 |  |  |  |  |
| Triglyceride | 0.480 |  |  |  |  |
| hsCRP | 0.434 |  |  |  |  |
| Cholesterol |  | 0.980 |  |  |  |
| LDL |  | 0.944 |  |  |  |
| A1c |  |  | 0.915 |  |  |
| GLU |  |  | 0.910 |  |  |
| SBP |  |  |  | 0.920 |  |
| DBP |  |  |  | 0.895 |  |
| METs (weekly) |  |  |  |  | 0.771 |
| Sitting time (minutes) |  |  |  |  | −0.718 |
| Eigen values | 2.522 | 1.972 | 1.939 | 1.791 | 1.166 |
| Rotation Sums of Squared Loadings (% of Variance) | 18.014 | 14.088 | 13.853 | 12.793 | 8.332 |
| Rotation Sums of Squared Loadings (Cumulative %) | 18.014 | 32.102 | 45.954 | 58.747 | 67.079 |
| BMI= body mass index;GLU=serum glucose; HDL=high density lipoprotein; LDL=low density lipoprotein; SBP=systolic blood pressure; DBP=diastolic blood pressure; MET= metabolic equivalent; HbA1C=hemoglobin A1C. | | | | | |
