# Supplementary material for: Factor analysis for the clustering of cardiometabolic risk factors and sedentary behavior, a cross-sectional study
Source: PLoS One. 2020 Nov 16;15(11):e0242365. doi: 10.1371/journal.pone.0242365 (PMC7668610; doi:10.1371/journal.pone.0242365)
Supplement: S4 Table — (DOCX) [file pone.0242365.s004.docx]

**S4 Table. Factor analysis in female patients**

|  | Component | | | | |
| --- | --- | --- | --- | --- | --- |
|  | 1 | 2 | 3 | 4 | 5 |
| BMI | 0.801 |  |  |  |  |
| Waist | 0.764 |  |  |  |  |
| HDL | −0.622 |  |  |  |  |
| hsCRP | 0.613 |  |  |  |  |
| Uric acid | 0.577 |  |  |  |  |
| Triglyceride | 0.468 |  |  |  |  |
| Cholesterol |  | 0.985 |  |  |  |
| LDL |  | 0.933 |  |  |  |
| GLU |  |  | 0.903 |  |  |
| A1c |  |  | 0.862 |  |  |
| DBP |  |  |  | 0.901 |  |
| SBP |  |  |  | 0.886 |  |
| METs (weekly) |  |  |  |  | 0.772 |
| Sitting time (minutes) |  |  |  |  | −0.699 |
| Eigen values | 2.641 | 2.007 | 1.862 | 1.841 | 1.155 |
| Rotation Sums of Squared Loadings (% of Variance) | 18.867 | 14.336 | 13.302 | 13.151 | 8.252 |
| Rotation Sums of Squared Loadings (Cumulative %) | 18.867 | 33.203 | 46.505 | 59.656 | 67.908 |
| BMI= body mass index;GLU=serum glucose; HDL=high density lipoprotein; LDL=low density lipoprotein; SBP=systolic blood pressure; DBP=diastolic blood pressure; MET= metabolic equivalent; HbA1C=hemoglobin A1C. | | | | | |
