# Supplementary material for: Factor analysis for the clustering of cardiometabolic risk factors and sedentary behavior, a cross-sectional study
Source: PLoS One. 2020 Nov 16;15(11):e0242365. doi: 10.1371/journal.pone.0242365 (PMC7668610; doi:10.1371/journal.pone.0242365)
Supplement: Attachment — Submitted filename: Response PONE-D-20-13875 R1 reviewer.docx [file pone.0242365.s006.docx]

**PONE-D-20-13875 R1 | Reviewer**

**Title: Factor analysis for the clustering of cardiometabolic risk factors and sedentary behavior, a cross-sectional study**

**Reviewing the revised manuscript | Response**

1. **Reviewer 1 wondered that there is a battery of studies showing that sedentary behavior clusters with factors of the metabolic syndrome, and claimed that knowledge gaps were not clearly explained.**
a. I agree with the reviewer.
b. Authors’ reply to this comment was not exactly relevant to the question.
c. In Response to this comment, the authors failed to appropriately revising the manuscript. They mentioned that “*There has been is a battery of studies demonstrating the association between sedentary and tradition CVD risk factors*”; however, the authors did not explain that association, and they did not give an example from a study examined such association to support the introductory statement (i.e., Ln 68-70). The authors were not clear if the phathophysiology of the association between sedentary behavior and CVD was previously explained or not. In addition, the authors in Ln 75-78 contrary the introductory statement where they stated “*few studies*”. Finally, the authors failed to state knowledge gaps, and then justify their study.
2. **Comment 2 of the reviewer 1, as well as comments 2 and 3 of the reviewer 2 was mainly about inclusion and exclusion criteria.**
a. Authors’ reply to this comment was not exactly relevant to the question. In general, the authors do not need to emphasize the self-paid issue. Instead, they need to clearly define the study population and study patients. In addition, they must clearly state inclusion and exclusion criteria, which were overlooked in the revised manuscript.
b. The revised manuscript (i.e., Ln 98-108) included not needed information, while overlooked important information such as the reason for selecting this healthcare center. Taking into account that self-paid reason for selecting this center is not relevant, unless this issue is matter in the analysis.
3. **Reviewer 1 asked the authors to explain the training and validation groups, and questioned the study population and study patients.**
a. I agree with the reviewer.
b. Authors’ response is not exactly relevant.
c. The authors need to define both training and validation groups in the current study, and the criteria used for selecting the subjects to such group.
d. Again, the authors need to clearly state inclusion and exclusion criteria.
4. **Reviewer 1 asked if the authors considered the method of machine learning in their methodology.**
a. Authors’ response is inconvenience.
b. In the revised manuscript, Ln 181-185 was not clear.
c. In the revised manuscript, authors explained some methodology for machine learning, whereas the current study considered factor analysis. Therefore, Ln 186-187 is not relevant to the current study method.
d. In the revised manuscript, Ln 188-193 neither relevant nor clear. The authors should justify classifying such study subjects as training group and others as validation group.
5. **In the comment 5 of the reviewer 1 and comment 4 of the reviewer 2, the analysis should consider the potential confounding effect of the age and sex.**

- The authors re-analyzed that correlation and provided supplemental tables.

6. **Reviewer 1 in the comment 6 claimed that the current manuscript lacks the novelty and explanation for relatively low variance for physical activity.**
a. The authors explained in their reply this issue; however, it was not clear in the revised manuscript. They need to clarify and state it in the revised manuscript.
b. In the reply, the authors mentioned that “A recent global heath observatory survey showed that 23% of men and 32% of women ≥18 years of age were insufficiently physically active. Over the past 15 years, the levels of insufficient activity did not improve (28.5% in 2001; 27.5% in 2016).” Does this apply to your country?
7. **Reviewer 2 wondered "*How do authors explain the low correlation between sitting time and total physical activity?*”**
a. I agree with the reviewer.
b. The authors need to make the revised as clear as their reply to this comment.
8. **Reviewer 1 claimed a huge limitation in this study.**

- The authors revised the manuscript, and added a section for study limitations.

**End**
