# Supplementary material for: Factor analysis for the clustering of cardiometabolic risk factors and sedentary behavior, a cross-sectional study
Source: PLoS One. 2020 Nov 16;15(11):e0242365. doi: 10.1371/journal.pone.0242365 (PMC7668610; doi:10.1371/journal.pone.0242365)
Supplement: Attachment — Submitted filename: Response to the reviwerPlosOneV4R.docx [file pone.0242365.s007.docx]

Point to point response to reviewers

**Response to reviewer 4**

***Regarding the comments: "In "Statistical analysis" section, it's written" categorical variables were expressed as the mean ± 95% confidence interval but it was expressed as number and percentage”.***

**Reply:** Thank you for your comments, we apologize for this incorrect statement. We have revised the statement in the method section to fit the presentation in the results section. (page 10 lines 10)

***Regarding the comments: "In the discussion section, It's written "Table 3 shows the baseline characteristics of all subjects according to physical activity". and "Indeed, as shown in Table 3, patients with low physical activity have multiple factors at the same time, making it difficult to evaluate the importance and correlation of individual risk factors". Here there are two remarks: first, the description of the results of the table are described in the result section and not the discussion section; Second, table 3 shows the Factor analysis of the training group and Table 5 shows the baseline characteristics of all subjects according to physical activity.”***

**Reply:** Thank you for your comments, we apologize for the mislabeling. We have moved the statement regarding table 3 to the discussion section and labeled the statements regarding the tables correctly in the revised manuscript.

(page 21 lines 4 to 6; page 34)

**Response to reviewer 1**

1. **Reviewer 1 wondered that there is a battery of studies showing that sedentary behavior clusters with factors of the metabolic syndrome, and claimed that knowledge gaps were not clearly explained.**
a. I agree with the reviewer.
b. Authors’ reply to this comment was not exactly relevant to the question.
c. In Response to this comment, the authors failed to appropriately revising the manuscript. They mentioned that “*There has been is a battery of studies demonstrating the association between sedentary and tradition CVD risk factors*”; however, the authors did not explain that association, and they did not give an example from a study examined such association to support the introductory statement (i.e., Ln 68-70). The authors were not clear if the phathophysiology of the association between sedentary behavior and CVD was previously explained or not. In addition, the authors in Ln 75-78 contrary the introductory statement where they stated “*few studies*”. Finally, the authors failed to state knowledge gaps, and then justify their study.

**Reply:** We appreciate this comment from reviewer 1 and apologize that our manuscript was not fully respond to the reviewer’s requirement. There are many studies reporting the association between sedentary behavior and various cardiovascular risk factors. For example, in the landmark NHANES 2003-2006 study, total sedentary time was detrimentally associated with several biomarkers including waist circumference, HDL-cholesterol, C-reactive protein, triglycerides, insulin, and insulin resistance. Breaks from long sitting period, independent of sedentary time, were beneficially associated with waist circumference, C-reactive protein, and fasting plasma glucose. However, while this study only demonstrated the close relationship between sedentary behavior and inflammation, other important risk factors such as total cholesterol, UA, HbA1C, were not evaluated in that study. Moreover, only a part of the study population has serum glucose measurement in this study. [8] In a later meta-analysis of more than 20000 subjects, Edwardson et al. demonstrated that patients with longer sedentary time have greater odds of having metabolic syndrome. However, metabolic syndrome represented a clinical syndrome with heterogenous characteristics and it is impossible to delineate the association between sedentary behavior and a particular component of metabolic syndrome from these evidences.[9] Thus, although sedentary behavior has been shown to be associated with many cardiovascular risk factors, the more detailed clustering relationship was unknown. Factor analysis has a unique advantage revealing the clustering structure of various risk factors. However, previous sedentary behavior rarely included sedentary behavior while those that did do not include major cardiovascular risk factors. Hence our study has the advantage to analyze the clustering structure of most used cardiovascular risk factors and sedentary behavior. We have revised our manuscript and added above descriptions in the section of Introduction. In addition, we have also added a statement about the pathophysiologic implication of sedentary behavior that sedentary behavior linked to reduced triglyceride metabolism, insufficient antioxidant production, and glucose intolerance in several animal studies. (page 5, lines 13 to page 6 line 13).

**References**

8.	HealyGN, MatthewsCE, DunstanDW, WinklerEAH, OwenN. Sedentary time and cardio-metabolic biomarkers in US adults: NHANES 200306. Eur Heart J. 2011;32: 590–597. doi:10.1093/eurheartj/ehq451

9.	EdwardsonCL, GorelyT, DaviesMJ, GrayLJ, KhuntiK, WilmotEG, et al. Association of Sedentary Behaviour with Metabolic Syndrome: A Meta-Analysis. O’ConnorKA, editor. PLoS One. 2012;7: e34916. doi:10.1371/journal.pone.0034916

2. **Comment 2 of the reviewer 1, as well as comments 2 and 3 of the reviewer 2 was mainly about inclusion and exclusion criteria.**
a. Authors’ reply to this comment was not exactly relevant to the question. In general, the authors do not need to emphasize the self-paid issue. Instead, they need to clearly define the study population and study patients. In addition, they must clearly state inclusion and exclusion criteria, which were overlooked in the revised manuscript.
b. The revised manuscript (i.e., Ln 98-108) included not needed information, while overlooked important information such as the reason for selecting this healthcare center. Taking into account that self-paid reason for selecting this center is not relevant, unless this issue is matter in the analysis.

**Reply:** We appreciate this comment from reviewer 1. We have removed some redundant description about the self-paid reason and added the following statement to describe our inclusion and exclusion criteria. “We included patients without significant symptoms or illness and excluded those who refused to participate, whose exam revealed an acute illness, or had a chronic condition that require regular follow up such as active cancer, heart failure, coronary artery disease or stroke. “(page 8, lines 6 to line 10 )

3. **Reviewer 1 asked the authors to explain the training and validation groups, and questioned the study population and study patients.**
a. I agree with the reviewer.
b. Authors’ response is not exactly relevant.
c. The authors need to define both training and validation groups in the current study, and the criteria used for selecting the subjects to such group.
d. Again, the authors need to clearly state inclusion and exclusion criteria.

**Reply:** We appreciate this instructive comment from reviewer 1. The training group and the validation group were randomly selected from the total study population. We have described the allocation process in the revised manuscript.

(page 13, lines 2 to line 3)

4. **Reviewer 1 asked if the authors considered the method of machine learning in their methodology.**
a. Authors’ response is inconvenience.
b. In the revised manuscript, Ln 181-185 was not clear.
c. In the revised manuscript, authors explained some methodology for machine learning, whereas the current study considered factor analysis. Therefore, Ln 186-187 is not relevant to the current study method.
d. In the revised manuscript, Ln 188-193 neither relevant nor clear. The authors should justify classifying such study subjects as training group and others as validation group.

**Reply:** We appreciate this comment from reviewer. We have added a short description about not considering machine learning methods in our study limitation. We will use machine learning method for the analysis of outcome data after the follow-ups of the VGH-HEALTHCARE study is completed. For the current study, we did not consider using machine learning in our methodology for two reasons. First, using machine learning technology for factor analysis has not been widely accepted because of lack of long-term outcome information. Second, our study purpose would like to investigate the percentage of variance of clustering cardiometabolic risk factors and the association between sedentary behavior and other associated risk factors.

(Page 23, line 5 to line 13)

5. **In the comment 5 of the reviewer 1 and comment 4 of the reviewer 2, the analysis should consider the potential confounding effect of the age and sex.**

- The authors re-analyzed that correlation and provided supplemental tables.

**Reply:** We appreciate this comment from the reviewer.

6. **Reviewer 1 in the comment 6 claimed that the current manuscript lacks the novelty and explanation for relatively low variance for physical activity.**
a. The authors explained in their reply this issue; however, it was not clear in the revised manuscript. They need to clarify and state it in the revised manuscript.
b. In the reply, the authors mentioned that “A recent global heath observatory survey showed that 23% of men and 32% of women ≥18 years of age were insufficiently physically active. Over the past 15 years, the levels of insufficient activity did not improve (28.5% in 2001; 27.5% in 2016).” Does this apply to your country?

Add novelty in revised manuscript

**Reply:** Thank you for your comment. As stated in our previous response, this study is the first to investigate the clustering relationship of a comprehensive array of cardiometabolic factors, including systemic inflammation, and sedentary information simultaneously in the general population of Taiwan. We also demonstrated that the physical activity factors do not cluster with traditional CV risk factors. Our results suggest that physical inactivity may exert its effect on cardiovascular disease in an independent and unique way. This result may prompt future researchers to explore the possible pathophysiologic mechanism behind the independent effect of the physical activity level. We have added the novelty of this study in the revised manuscript. In response to your second comment, inadequate physical activity was observed in the Taiwanese population as well.[42] Although our data is not a good representative of all Taiwanese people, the percentage is reasonable for the Taiwanese general population. There may be a selection bias, that we cannot avoid selecting subjects with better higher social economic status whose sedentary behavior may be different from other population. (page 17, lines 11 to line 17)

**References:**

42.	WuX, TsaiSP, TsaoCK, ChiuML, TsaiMK, LuPJ, et al. Cohort Profile: The Taiwan MJ Cohort: Half a million Chinese with repeated health surveillance data. Int J Epidemiol. 2017;46: 1744-1744g. doi:10.1093/ije/dyw282

7. **Reviewer 2 wondered "*How do authors explain the low correlation between sitting time and total physical activity?*”**
a. I agree with the reviewer.
b. The authors need to make the revised as clear as their reply to this comment.

**Reply:** In our previous response to reviewers, we have addressed this issue by stating that traditionally, sedentary behavior and total physical activity are believed to be independent risk factors for cardiovascular events [6,7]. However, the association between physical activity and sedentary behavior is not consistent. Some studies reported no significant association between the two while others showed that there was some association [43]. This discrepancy can be explained by the fact that a person can be both sedentary and physically active (the Active Couch Potato phenomenon, describes someone who meets the recommendations for physical activity but still sits around for long periods of the day.) [44]. In this study, we used the IPAQ questionnaire, which is a widely validated questionnaire that is recommended by the WHO, to evaluate the association between sedentary behavior and weekly physical activity [45,46]. We believe that the low but still significant correlation between sitting time is consistent with previous studies, and it can be explained by the fact that non-sedentary physical activity in the general population is quite variable. We believe our original description should be sufficient.

**References:**

6.	PandeyA, SalahuddinU, GargS, AyersC, KulinskiJ, AnandV, et al. Continuous dose-response association between sedentary time and risk for cardiovascular disease a meta-analysis. JAMA Cardiol. 2016;1: 575–583. doi:10.1001/jamacardio.2016.1567

7.	EkelundU, BaumanA, LeeIM. Effects of early physical exercise on later health – Authors’ reply. The Lancet. Lancet Publishing Group; 2017. p. 801. doi:10.1016/S0140-6736(17)30506-8

43.	SilfeeV, LemonS, LoraV, RosalM. Sedentary behavior and cardiovascular disease risk factors among latino adults. J Health Care Poor Underserved. 2017;28: 798–811. doi:10.1353/hpu.2017.0075

44.	OwenN, HealyGN, MatthewsCE, DunstanDW. Too much sitting: The population health science of sedentary behavior. Exerc Sport Sci Rev. 2010;38: 105–113. doi:10.1097/JES.0b013e3181e373a2

45.	CraigCL, MarshallAL, SjöströmM, BaumanAE, BoothML, AinsworthBE, et al. International physical activity questionnaire: 12-Country reliability and validity. Med Sci Sports Exerc. 2003;35: 1381–1395. doi:10.1249/01.MSS.0000078924.61453.FB

46.	BoonRM, HamlinMJ, SteelGD, RossJJ. Validation of the New Zealand physical activity questionnaire (NZPAQ-LF) and the international physical activity questionnaire (IPAQ-LF) with accelerometry. Br J Sports Med. 2010;44: 741–746. doi:10.1136/bjsm.2008.052167

8. **Reviewer 1 claimed a huge limitation in this study.**

- The authors revised the manuscript, and added a section for study limitations.

**Reply:** We appreciate your comment.
